# Endoscopic features and clinical outcomes of cytomegalovirus gastroenterocolitis in immunocompetent patients

**DOI:** 10.1038/s41598-021-85845-8

**Published:** 2021-03-18

**Authors:** Jiyoung Yoon, Junghwan Lee, Dae Sung Kim, Jin Wook Lee, Seung Wook Hong, Ha Won Hwang, Sung Wook Hwang, Sang Hyoung Park, Dong-Hoon Yang, Byong Duk Ye, Seung-Jae Myung, Hwoon-Yong Jung, Suk-Kyun Yang, Jeong-Sik Byeon

**Affiliations:** Department of Gastroenterology, Asan Medical Center, University of Ulsan College of Medicine, 88 Olympic-ro 43-gil, Songpa-gu, Seoul, 05505 South Korea

**Keywords:** Diseases, Gastroenterology

## Abstract

We aimed to investigate the endoscopic features and clinical course of CMV gastroenterocolitis in immunocompetent patients. We reviewed the medical records and endoscopic images of 86 immunocompetent patients with CMV gastroenterocolitis. Immunocompetent patients were defined as those without congenital or acquired immunodeficiency syndrome, use of anti-cancer chemotherapeutic and immunosuppressive agents, and inflammatory bowel diseases. The mean age was 65.5 ± 11.8 years and 53 (61.6%) were male. Sixty-eight (79.1%) patients had comorbidities. Upper gastrointestinal-dominant, small bowel-dominant, and colon-dominant types were observed in 19, 7, and 60 patients, respectively. Endoscopic features could be classified into discrete ulcerative type with/without exudate and diffuse erythematous type with/without exudate. Antiviral treatment with ganciclovir was initiated in 51 patients (59.3%), 40 of whom improved and 1 improved after changing ganciclovir to foscarnet. Thirty-three patients (38.4%) improved without antiviral treatment. Surgery was necessary in two patients because of colon perforation before antiviral treatment. Another two patients underwent surgery because of sigmoid stricture and cecal perforation during antiviral treatment. Endoscopic type was not associated with clinical outcomes, such as surgery and death. CMV gastroenterocolitis in immunocompetent patients mostly occur in older patients with comorbidities, and the endoscopic features vary with no association with clinical outcomes.

## Introduction

Cytomegalovirus (CMV) is a common virus that reportedly infects more than half of all adults by age 40 years. It is estimated that 40–100% of immunocompetent adults worldwide are CMV seropositive^1^. Like other herpes viruses, CMV remains in a dormant phase for the entire life of an infected individual after the resolution of the initial infection episode. This state of latency allows CMV to reactivate when the host’s immunity becomes compromised, such as in the background of acquired immunodeficiency syndrome, organ transplantation, and malignancy treated with chemotherapy^1,2^. CMV diseases in immunocompromised patients can affect several organs, such as the lung, retina, and brain^3^. The gastrointestinal (GI) tract is also one of the most common organs of CMV disease development. CMV gastroenterocolitis manifests with various symptoms, including dysphagia, abdominal pain, diarrhea, and GI bleeding. The endoscopic findings are nonspecific, with mucosal ulceration being the most common^4^.

Although rare, CMV diseases caused by reactivation of CMV have been recognized in patients without typical immunocompromised conditions, especially in older patients with chronic diseases, such as end-stage renal disease, diabetes mellitus, and coronary artery disease^2–8^. Despite the lower morbidity and mortality of CMV diseases in immunocompetent hosts than in immunocompromised patients, life-threatening situations have been described, especially in older patients and in those with critical illness and/or comorbidities^9,10^. Nonetheless, because of its rarity in immunocompetent hosts, the clinical manifestations and endoscopic features are not clearly understood. Thus, timely suspicion and early diagnosis of CMV gastroenterocolitis in immunocompetent patients are challenging. In addition, antiviral treatment responses are yet to be clarified and there is no consensus on the necessity of antiviral treatment in CMV gastroenterocolitis in immunocompetent patients.

The purpose of this study was to investigate the clinical presentation, endoscopic findings, and clinical outcomes of CMV gastroenterocolitis in immunocompetent patients, thereby helping gastroenterologists in making timely diagnosis of and achieving the best treatment outcomes for CMV gastroenterocolitis in immunocompetent patients.

## Results

### Baseline characteristics

**Figure 1:**
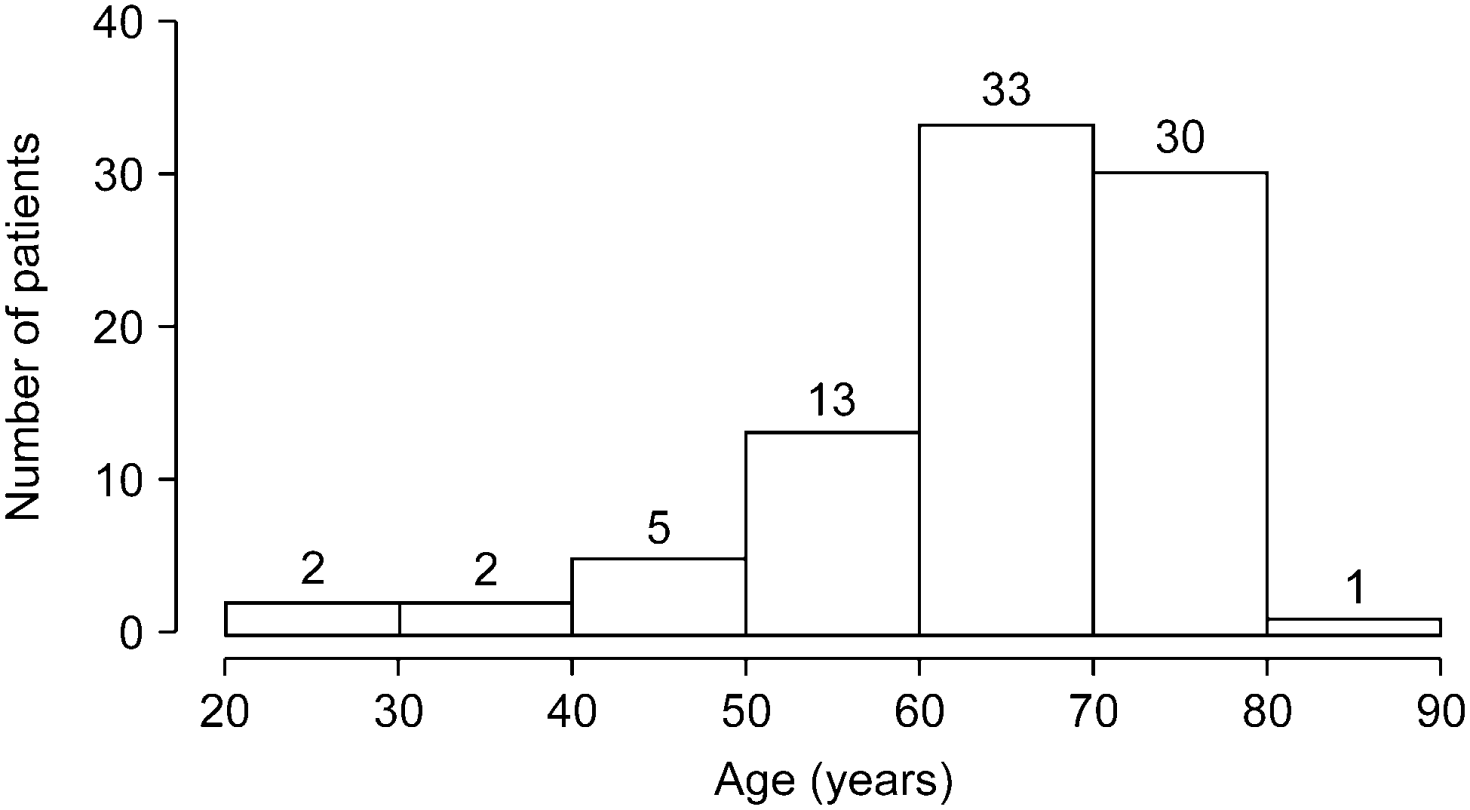
Age at the diagnosis of cytomegalovirus gastroenterocolitis in immunocompetent patients.

The mean and median age of the 86 patients included in this study were 65.5 ± 11.8 years and 68.0 (60.2–74.0) years, respectively. Sixty-four patients (74.4%) were aged > 60 years at the diagnosis of CMV gastroenterocolitis, with 33 patients in their 60 s and 30 patients in their 70 s (Fig. 1). Of the 86 total patients, 53 were men and 33 were women. Among them, 68 (79.1%) had preexisting chronic diseases, such as diabetes mellitus, chronic kidney disease, and cardiovascular diseases. Thirty-six patients (41.9%) with underlying chronic diseases also had comorbid acute illnesses, such as pneumonia, urinary tract infection, and intraabdominal abscess. Eighteen patients (20.9%) were previously healthy without both chronic diseases and acute illnesses. Concurrent extra-GI CMV diseases were not detected in any patients. The baseline characteristics of the patients are summarized in Table 1.

**Table 1:** Baseline characteristics of immunocompetent patients with cytomegalovirus gastroenterocolitis.

	CMV gastroenterocolitis in immunocompetent patients (N = 86)
Age, years, median (IQR)	68.0 (60.2–74.0)
Male sex, n (%)	53 (61.6%)
**ASA classification at presentation, n (%)**	
Class 1	7 (8.1%)
Class2	31 (36.0%)
Class 3	30 (34.9%)
Class 4	18 (21%)
BMI^a^, kg/m^2^, median (IQR)	22.6 (20.6–24.5)
**Underlying chronic disease, n (%)**	68 (79.1%)
Diabetes mellitus	34 (39.5%)
Hypertension	31 (36.0%)
Cardiovascular disease	21 (24.4%)
End-stage renal disease on dialysis	15 (17.4%)
Other chronic diseases^b^	42 (48.8%)
No underlying chronic disease^c^	18 (20.9%)
**Other risk factors**	
Acute illness caused by ongoing infection^d^	36 (41.9%)
ICU care	34 (39.5%)
Antibiotic use within 1 month	66 (76.7%)
Transfusion within 1 month	42 (48.8%)
**Location of endoscopic involvement, n (%)**^**e**^	
UGI-dominant	19 (22.1%)
UGI only	16 (18.6%)
UGI + SB	2 (2.3%)
UGI + colon	1 (1.2%)
SB-dominant	7 (8.1%)
SB only	4 (4.6%)
SB + UGI	2 (2.3%)
SB + colon	1 (1.2%)
Colon-dominant	60 (69.8%)
Colon only	53 (61.6%)
Colon + UGI	6 (6.9%)
Colon + SB	1 (1.2%)

CMV, cytomegalovirus; IQR, interquartile range; ASA, American Society of Anesthesiologists; BMI, body mass index; ICU, intensive care unit; SD, standard deviation; UGI, upper gastrointestinal (esophagus and stomach); SB, small bowel (duodenum, jejunum, and ileum).

^a^BMI data were not available for 2 of the 86 patients.

^b^Other chronic diseases included chronic kidney disease, chronic liver disease, neurologic disease, and rheumatologic disease.

^c^These 18 patients did not have both underlying chronic diseases and ongoing acute illnesses, such as infection.

^d^Ongoing infection included pneumonia, urinary tract infection, diabetes mellitus foot, liver abscess, sepsis, infective endocarditis, intraabdominal abscess, and muscle abscess.

^e^The total number of involved segments was 99.

### Endoscopic location of involvement

We classified the endoscopic location of involvement as upper GI (UGI)-dominant, small bowel (SB)-dominant, and colon-dominant, according to the GI segment mainly involved in CMV diseases. The UGI included the esophagus and stomach, whereas SB included the duodenum, jejunum, and ileum. Nineteen patients (22.1%) were included in the UGI-dominant group, 7 (8.1%) in the SB-dominant group, and 60 (69.8%) in the colon-dominant group. Thirteen patients showed endoscopic involvement of two or more GI segments, such as the colon plus the UGI and the colon plus the SB.

The UGI-, SB-, and colon-dominant groups did not show any differences in terms of age, sex, ASA class, and presence or absence of underlying chronic diseases. Body mass index was the lowest in the UGI group. The initial presenting symptoms varied according to the endoscopic location (*P* < 0.001). The UGI-dominant group more often presented with nausea/vomiting, odynophagia, and hematemesis. The most common symptom of the SB-dominant group was abdominal pain (five of seven patients, 71.4%). Hematochezia was the most common symptom in the colon-dominant group (33 of 60 patients, 55.0%). Serum albumin level was significantly different among the three groups, with the SB-dominant group showing the lowest value and the UGI-dominant group showing the highest value (1.9 ± 0.6 vs. 3.0 ± 0.8 g/dL, *P* = 0.002). The positive rates of both serum CMV PCR and CMV antigenemia were not different among the UGI-, SB-, and colon-dominant groups. The overall positive rates of serum CMV PCR and CMV antigenemia were 54.3% (25 of 46) and 35.7% (20 of 56), respectively. The clinical features of the UGI-, SB-, and colon-dominant groups are summarized in Table 2.

**Table 2:** Clinical characteristics of cytomegalovirus gastroenterocolitis according to the endoscopic location of involvement.

	UGI-dominant group (n = 19)	SB-dominant group (n = 7)	Colon-dominant group (n = 60)	*P* value
Age, years, median (IQR)	68.0 (59.5–70.5)	70.0 (63.5–78.0)	67.0 (61.7–74.2)	0.553
Male sex, n (%)	10 (52.6%)	6 (100.0%)	37 (61.7%)	0.506
**ASA classification at presentation, n (%)**				0.133
Class 1	3 (15.8)	1 (14.3)	3 (5.0)	
Class 2	5 (26.3)	6 (85.7)	20 (33.3)	
Class 3	7 (36.8)	0 (0.0)	23 (38.3)	
Class 4	4 (21.1)	0 (0.0)	14 (23.4)	
BMI^a^, kg/m^2^, median (IQR)	21.2 (19.3–23.1)	24.0 (22.3–25.3)	22.7 (20.6–24.8)	0.039
Presence of underlying chronic disease	12 (63.2%)	6 (85.7%)	49 (81.7%)	0.208
**Other risk factors**				
Ongoing infection	6 (31.6%)	1 (14.3%)	29 (48.3%)	0.132
ICU care	4 (21.1%)	1 (14.3%)	29 (48.3%)	0.038
Antibiotic use < 1 month	12 (63.2%)	5 (83.3%)	48 (80.0%)	0.268
Transfusion < 1 month	9 (47.4%)	3 (42.9%)	30 (50.0%)	0.928
**Symptoms, n (%)**				< 0.001
Abdominal pain	2 (10.5)	5 (71.4)	13 (21.7)	
Melena	4 (21.1)	1 (14.3)	2 (3.3)	
Hematochezia	0 (0.0)	1 (14.3)	33 (55.0)	
Diarrhea	0 (0.0)	0 (0.0)	12 (20.0)	
Hematemesis	2 (10.5)	0 (0.0)	0 (0.0)	
Odynophagia	3 (15.8)	0 (0.0)	0 (0.0)	
Nausea/vomiting	7 (36.8)	0 (0.0)	0 (0.0)	
No symptom	1 (5.3)	0 (0.0)	0 (0.0)	
**Laboratory findings**				
WBC, /μL	8100.0 ± 3703.9	11,000.0 ± 4316.5	9269.5 ± 4621.7	0.334
Hemoglobin, g/dL	10.4 ± 2.0	11.6 ± 2.5	10.1 ± 1.8	0.167
Platelet, × 10^3^/μL	245.6 ± 146.2	299.6 ± 131.1	278.3 ± 136.9	0.594
Albumin, g/dL	3.0 ± 0.8	1.9 ± 0.6	2.5 ± 0.7	0.002
CRP, mg/dL	4.0 ± 7.2	3.8 ± 2.3	4.8 ± 4.8	0.822
Positive CMV PCR (serum) (n = 46)	5/10 (50%)	6/7 (85.7%)	14/29 (48.2%)	0.800
Positive CMV antigenemia (serum) (n = 56)	3/10 (30%)	1/4 (25%)	16/42 (38.0%)	0.577

CMV, cytomegalovirus; BMI, body mass index; WBC, white blood cell; CRP, C-reactive protein; PCR, polymerase chain reaction.

^a^Body mass index data were not available for 2 of the 86 patients.

### Endoscopic types according to gross features

**Figure 2:**
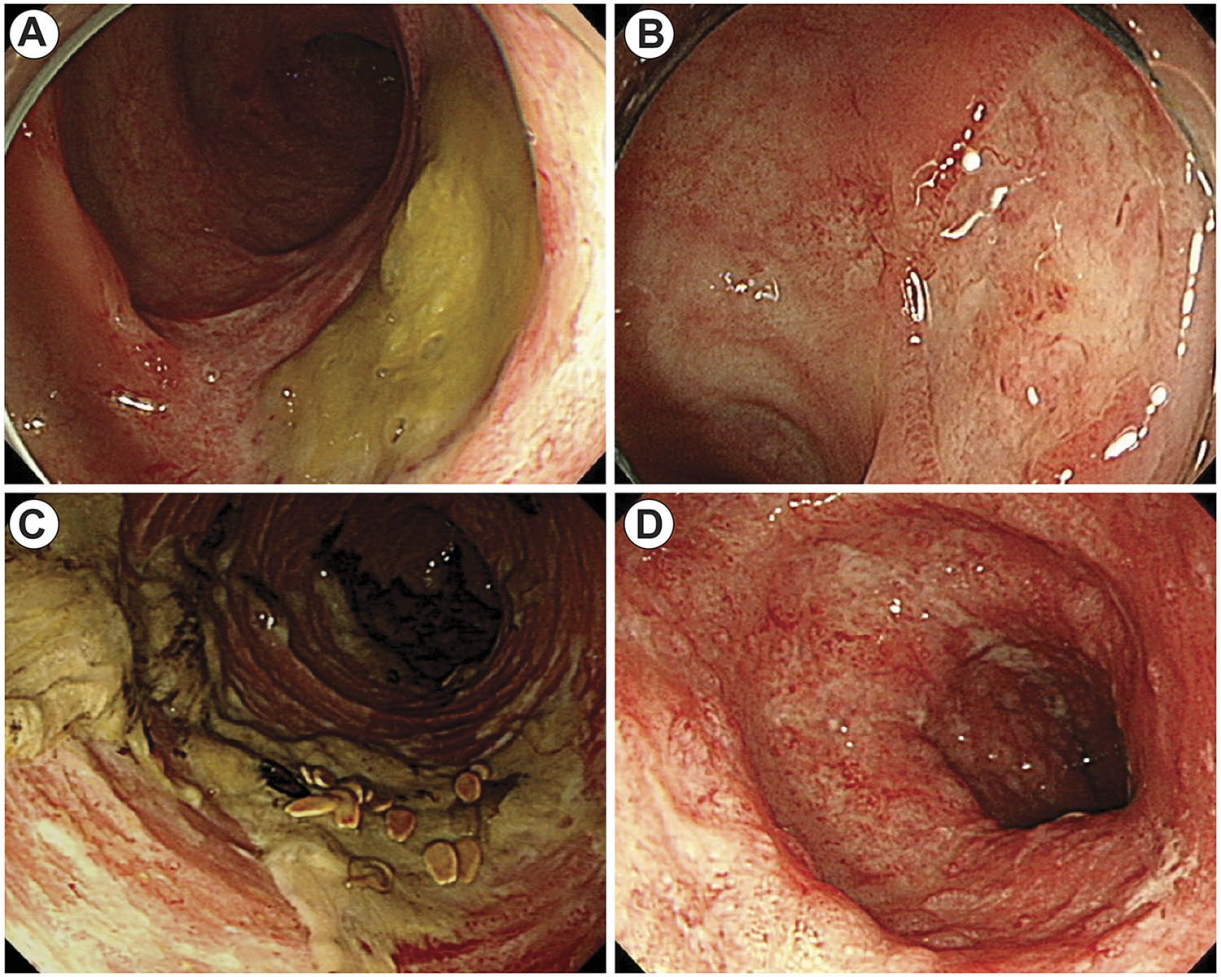
Endoscopic types according to gross features. (**A**) Discrete ulcerative type with exudate. (**B**) Discrete ulcerative type without exudate. (**C**) Diffuse erythematous type with exudate. (**D**) Diffuse erythematous type without exudate. All images (**A**–**D**) are cytomegalovirus colitis.

The endoscopic gross features were classified into the discrete ulcerative type with or without exudate and the diffuse erythematous type with or without exudate (Fig. 2). The morphology of ulcers in the discrete ulcerative type widely varied in terms of size and shape. The diffuse erythematous type was assigned if erythema, swelling, loss of submucosal vascularity, and friability were the dominant features without definite ulcers. More typical images of the four endoscopic types are presented in Supplementary Figure 1.

**Figure 3:**
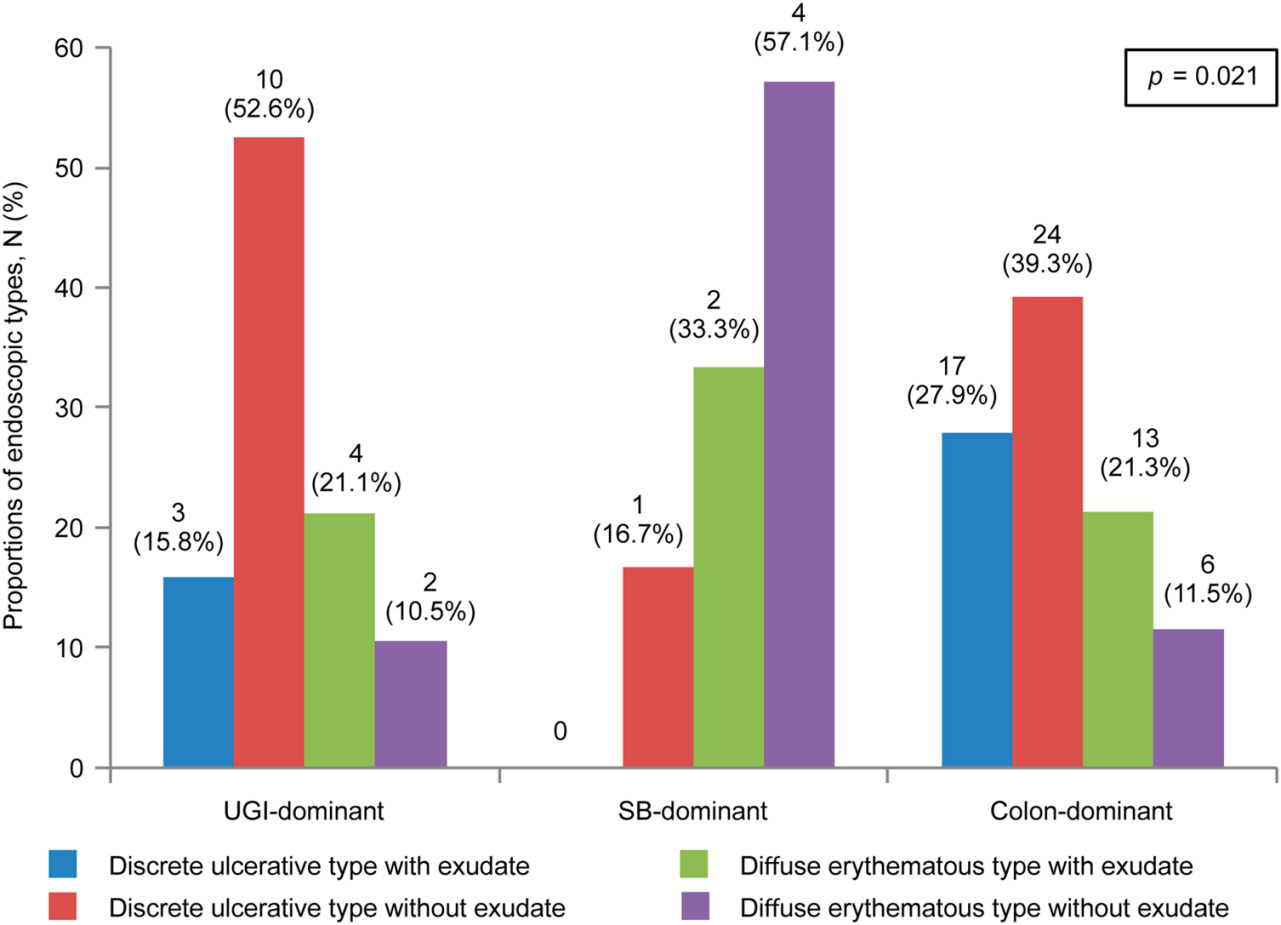
Proportions of endoscopic types according to sites of cytomegalovirus involvement. UGI, upper gastrointestinal tract; SB, small bowel.

The endoscopic types varied according to the location CMV involvement (*P* = 0.021) (Fig. 3). The discrete ulcerative type without exudate was the most common endoscopic type in the UGI-dominant group (10 of 19 patients, 52.6%). Ulcerative types were rare and diffuse erythematous types with or without exudate were common in the SB-dominant group. The colon-dominant group most commonly showed the discrete ulcerative type without exudate (24 of 60 patients, 39.3%).

Table 3 presents the clinical data analyzed according to the four endoscopic types. Patients with the diffuse erythematous type with exudate were significantly older than those with other endoscopic types (*P* = 0.005). The endoscopic biopsy yield was not different among the four endoscopic types. The positivity of CMV PCR and immunohistochemical staining in biopsy tissues was similarly high (69.4–100.0%) among all four endoscopic types. In comparison, CMV inclusion bodies were less commonly detected in biopsy tissues in all four endoscopic types (11.7–41.6%).

**Table 3:** Clinical characteristics of cytomegalovirus gastroenterocolitis according to endoscopic types.

	Discrete ulcerative type with exudate (n = 20)^a^	Discrete ulcerative type without exudate (n = 35)^a^	Diffuse erythematous type with exudate (n = 19)^a^	Diffuse erythematous type without exudate (n = 12)^a^	*P* value
Age, years, median (IQR)	68.0 (65.0–73.0)	66.0 (53.5–68.0)	74.0 (68.0–77.0)	70.0 (64.2–75.0)	0.005
Male sex, n (%)	11 (55.0)	20 (57.1)	12 (63.2)	10 (83.3)	0.380
**ASA classification at presentation, n (%)**					0.985
Class 1	2 (10.0)	3 (8.6)	1 (5.3)	1 (8.3)	
Class 2	6 (30.0)	13 (37.1)	7 (36.8)	5 (41.7)	
Class 3	7 (35.0)	12 (34.3)	6 (31.6)	5 (47.7)	
Class 4	5 (25.0)	7 (20.0)	5 (26.3)	1 (8.3)	
BMI^b^, kg/m^2^, median (IQR)	22.1 (20.2–23.3)	22.7 (20.2–23.8)	23.3 (21.1–24.6)	23.6 (22.0–25.7)	0.385
**Tissue biopsy**^**c**^					
CMV PCR (+), n = 84	19/23 (82.6%)	26/32 (81.21%)	15/20 (75.0%)	7/9 (77.7%)	0.927
CMV IHC (+), n = 91	17/23 (73.9%)	25/36 (69.4%)	18/21 (85.7%)	11/11 (100.0%)	0.133
CMV inclusion body (+), n = 93	2/17 (11.7%)	8/25 (32%)	5/22 (22.7%)	5/12 (41.6%)	0.296

CMV, cytomegalovirus; IQR, interquartile range; ASA, American Society of Anesthesiologists; BMI, body mass index; PCR, polymerase chain reaction; IHC, immunohistochemistry.

^a^The endoscopic types of patients were defined according to the main involved location.

^b^BMI data were not available for 2 of the 86 patients.

^c^The number of tissue biopsy results were based not on the number of patients but on the number of involved gastrointestinal segments.

### Clinical course of CMV gastroenterocolitis in immunocompetent patients

**Figure 4:**
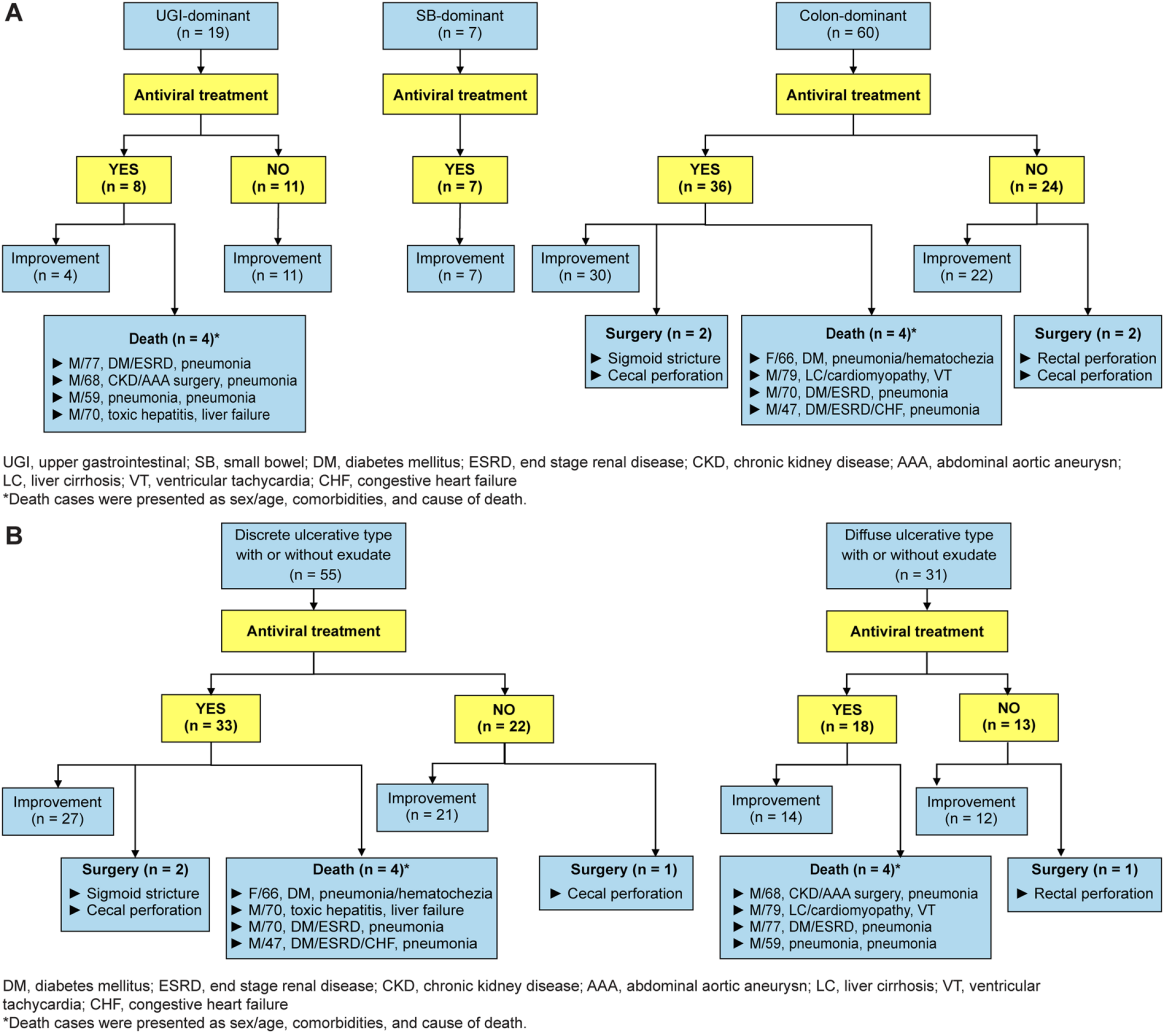
Clinical course of cytomegalovirus gastroenterocolitis in immunocompetent patients according to (**A**) location of involvement. (**B**) endoscopic type. UGI, upper gastrointestinal; SB, small bowel; DM, diabetes mellitus; ESRD, end-stage renal disease; CKD, chronic kidney disease; AAA, abdominal aortic aneurysm; LC, liver cirrhosis; VT, ventricular tachycardia; CHF, congestive heart failure. *Death cases are presented as sex/age, comorbidities, and cause of death.

Antiviral therapy with intravenous ganciclovir was administered as an initial treatment in 51 patients (59.3%). Of these 51 patients, 40 (78.4%) improved with a mean treatment duration of 17.1 ± 5.9 days. One patient with CMV ileitis who did not improve with ganciclovir eventually improved after rescue therapy with foscarnet. Two patients underwent surgical colectomy because of cecal perforation and sigmoid stricture, respectively, during antiviral therapy with ganciclovir. Eight patients died during antiviral therapy with ganciclovir, seven of whom died of active comorbid conditions, such as pneumonia and liver failure, and one died of aggravating pneumonia plus GI bleeding from CMV colitis (Fig. 4).

The other 35 of the 86 patients did not receive antiviral therapy because (1) their symptoms improved with supportive care before and around the histologic confirmative diagnosis of CMV gastroenterocolitis in 33 patients, or (2) emergency surgeries were performed in two patients with CMV colitis for cecal and rectal perforations, respectively, before the histologic diagnosis of the endoscopy biopsy specimens. The UGI-dominant group did not receive antiviral therapy more frequently (11 of 19 patients, 57.9%) than the SB-dominant group (0 of 7 patients, 0%) and the colon-dominant group (24 of 60 patients, 40.0%) (*P* = 0.028). Most UGI-dominant patients who did not receive antiviral therapy improved with proton pump inhibitors.

### Risk factors for poor clinical outcomes

In the univariable analysis, C-reactive protein (CRP) was associated with the necessity of surgery (OR 1.14, 95% confidence interval [CI] 1.03–1.26; *P* = 0.044). CRP was an independent risk factor for surgery in the multivariable analysis (OR 1.22, 95% CI 1.05–1.41; *P* = 0.009) (Supplementary Table 1).

In the univariable analysis, ASA class, hemoglobin, CRP, albumin, and intensive care unit stay were associated with in-hospital mortality in immunocompetent patients with CMV gastroenterocolitis. CRP was an independent risk factor for in-hospital mortality in the multivariable analysis (OR 1.22, 95% CI 1.06–1.34; *P* = 0.005) (Table 4).

**Table 4:** Risk factors for in-hospital mortality in immunocompetent patients with cytomegalovirus gastroenterocolitis.

	Univariable analysis		Multivariable analysis	
	OR (95% CI)	*P* value	OR (95% CI)	*P* value
Age	1.01 (0.95–1.09)	0.699		
Male sex	4.87 (0.57–41.53)	0.148		
BMI^a^	1.05 (0.85–1.29)	0.663		
ASA class	3.26 (1.24–8.57)	0.016		
**Location**				
UGI-dominant group	3.73 (0.83–16.71)	0.085		
SB-dominant group	∞	1.000		
Colon-dominant group	1 (ref)	0.091		
**Laboratory findings**				
Hemoglobin	0.5 (0.28–0.9)	0.020	0.63 (0.37–1.08)	0.092
CRP	1.19 (1.05–1.34)	0.005	1.22 (1.06–1.34)	0.005
Albumin	0.65 (0.22–1.89)	0.428		
**Endoscopic types**				
Discrete ulcer with or without exudate	1.00			
Diffuse erythema with or without exudate	1.89 (0.44–8.15)	0.394		
**Other risk factors**				
Ongoing infection	2.53 (0.56–11.34)	0.226		
ICU stay	5.36 (1.01–28.34)	0.048	2.92 (0.05–2.92)	0.341
Transfusion within 1 month	8.6 (1.01–73.26)	0.049		
Antiviral therapy	∞	1.000		

OR, odds ratio; CI, confidence interval; BMI, body mass index; ASA, American Society of Anesthesiologists; UGI, upper gastrointestinal tract; SB, small bowel; CRP, C-reactive protein; ICU, intensive care unit.

^a^BMI data were not available for 2 of the 86 patients.

## Discussion

In this descriptive analysis, we found that most of the immunocompetent patients diagnosed with CMV gastroenterocolitis were old and had underlying chronic diseases with or without ongoing acute illnesses. Of the whole GI tract, the colon was the most frequently involved segment. Endoscopic features could be classified into the ulcerative and erythematous types with or without exudate, which were not associated with clinical outcomes. Most patients improved with antiviral therapy and some patients improved with only supportive care. However, adverse events, such as perforation and stricture occurred in a minority of patients with CMV colitis. In-hospital mortality occurred in a minority of patients, although the causes of death were not CMV gastroenterocolitis but ongoing active comorbidities in most cases.

Although we found that CMV gastroenterocolitis could occur in immunocompetent patients, the issue of whether the patients were truly immunocompetent is still debated. Most patients were old and had chronic and/or acute comorbidities, which is comparable to previous reports^3,8,11^. It is well known that immune dysfunction followed by an increased risk of infection may be associated with chronic comorbidities, such as diabetes mellitus and chronic kidney disease^12,13^. Impaired immunity, including compromised mucosal and secretory immune response in the GI tract has also been demonstrated in older patients^14^. Taking these evidences together with the median age of 68 years, presence of comorbidities in 79.1% of patients in our study, and similar results from previous reports^8,15^, we suggest that CMV gastroenterocolitis may be rare in truly immunocompetent hosts who are young; and most patients may have risk factors related to impaired immunity, such as old age and comorbidities, although they do not have typical immunocompromised conditions, such as congenital or acquired immunodeficiency syndrome and use of chemotherapeutic and/or immunosuppressive agents.

The colon was the most frequently involved site in immunocompetent patients, similar to that reported in previous studies^3^. The most common symptom of CMV colitis in immunocompetent patients was GI bleeding, such as hematochezia and melena, followed by abdominal pain and diarrhea, also similar to those reported earlier^8,15,16^. The frequent symptoms differed according to the main involved sites in our study. Nausea and/or vomiting were the most common symptoms in the UGI-dominant group, and abdominal pain was the most common symptom in the SB-dominant group. Systemic symptoms, such as fever and malaise, were rare. This suggests that clinical presentation may depend not on CMV infection itself but on the main involved site.

The endoscopic features of CMV gastroenterocolitis in immunocompetent patients vary. In a retrospective study of immunocompetent patients diagnosed with CMV colitis, the most common endoscopic abnormalities were well-demarcated ulcerations (50%) followed by ulceroinfiltrative changes (25%) and pseudomembrane formation (25%)^17^. We classified the endoscopic findings into the discrete ulcerative type with or without exudate and the diffuse erythematous type with or without exudate. As in the previous study^17^, the ulcerative types were predominant (55 of 86 patients, 64%). In addition, thick pseudomembrane-like exudate was also common, occurring in 36.4% (20 of 55 patients) in the discrete ulcerative type and in 61.3% (19 of 31 patients) in the diffuse erythematous type. Interestingly, two patients were diagnosed with CMV enteritis limited to the jejunum and ileum by enteroscopy, which showed prominent, thick, pseudomembrane-like exudate covering nearly the whole mucosa without definite ulcers (Supplementary Figure 1H and I). Thus, endoscopists should be aware of the various endoscopic features, including thick exudates, to have a high index of suspicion for CMV gastroenterocolitis even in immunocompetent patients.

The presence of CMV in the GI mucosal tissue was identified in 75.0%–82.6% by CMV PCR and in 69.4–100.0% by CMV immunohistochemical staining (Table 3). In contrast, the presence of CMV in the blood was identified only in 35.7% by CMV antigenemia and in 54.3% by CMV PCR, which is similar to the 38.6% positive serologic results reported in a previous meta-analysis of CMV colitis in immunocompetent patients^3^. This suggests that CMV gastroenterocolitis in immunocompetent patients may be a local reactivation rather than a systemic reactivation, and that tissue CMV PCR may be a more useful tool for the diagnosis of CMV gastroenterocolitis in immunocompetent patients^18^.

Although in-hospital mortality occurred in 8 (9.3%) patients in our study, the causes of death were aggravation of underlying chronic and/or acute comorbidities in all patients except in one patient whose cause of death was comorbid illness plus GI bleeding from CMV colitis. Thus, we suggest that the prognosis of CMV gastroenterocolitis itself in immunocompetent patients may be generally favorable. Of the 51 patients who were treated with ganciclovir, 40 patients improved, and additionally, 1 patient also improved with foscarnet treatment after ganciclovir failure, indicating a response rate of 80.4% by medical treatment with antiviral agents. This finding suggests the high effectiveness of antiviral therapy in the management of CMV gastroenterocolitis in immunocompetent patients, which is compatible with a previous study that showed that treatment with antiviral agents was a significant protective factor against death^16^. Interestingly, 33 of the 86 patients (38.4%) improved without specific therapies, such as antiviral treatment and surgery. The attending physician did not prescribe antiviral agents for these patients because the clinical symptoms and signs improved without a specific therapy before and around the histologic confirmative diagnosis of CMV gastroenterocolitis. Thus, we cautiously suggest that close observation with supportive care could be an alternative management option for CMV gastroenterocolitis, especially in the UGI-dominant group of immunocompetent patients, if the patients are clinically stable and symptoms improve with supportive care only. Otherwise, antiviral treatment should be the treatment of choice in most cases, especially in patients with high CRP levels because CRP was an independent risk factor for poor clinical courses, such as the need for surgery and in-hospital mortality.

Stricture and perforation developed in one (1.2%) and three (3.5%) patients, respectively. Therefore, we suggest that gastroenterologists should be aware of the possibility of adverse events in immunocompetent patients with CMV gastroenterocolitis although the incidence rate is low. Specifically, gastroenterologists may consider early rapid empirical antiviral treatment if the endoscopic features suggest a possibility of severe CMV gastroenterocolitis, because perforation occurred before the start of antiviral treatment in two patients waiting for the biopsy results in our study.

The present study had several limitations. First, this was a single-center retrospective analysis, which limits the generalizability of our findings. Second, patient management was based not on a standardized protocol but on the judgement of the attending physicians, which hinders making a confirmative conclusion about the effect of the treatment algorithm. Third, although CRP was identified as a risk factor for surgery and in-hospital mortality, the confidence level of this finding was not high because of the small number of patients who underwent surgery or died. Nonetheless, we believe that our study is meaningful because, to our knowledge, this is the largest case series of CMV gastroenterocolitis in immunocompetent patients that investigated not only clinical findings but also endoscopic features, which were analyzed in association with clinical outcomes.

In conclusion, CMV gastroenterocolitis can develop in patients without typical immunocompromised conditions, especially in older patients with comorbidities. Gastroenterologists should be aware of the various endoscopic features of CMV gastroenterocolitis in immunocompetent patients so that timely diagnosis and treatment could be performed.

## Materials and methods

### Patients

We included all immunocompetent adult patients who were diagnosed with CMV gastroenterocolitis at Asan Medical Center, Seoul, Korea, from January 2000 to April 2018. Immunocompetent patients were defined as those without typical immunocompromised conditions; i.e., patients who did not have congenital or acquired immunodeficiency syndrome, solid organ transplantation, hematopoietic stem cell transplantation, malignancies treated with chemotherapy, and those who did not take immunosuppressive agents, including steroids (20 mg/day of prednisolone or equivalent), for > 2 weeks. Because CMV enterocolitis is not uncommon in long-standing inflammatory bowel diseases, patients with inflammatory bowel diseases were also excluded from immunocompetent patients despite not having typical immunocompromised conditions. In this study, a diagnosis of CMV gastroenterocolitis was made if patients met both of the following criteria: (1) presence of macroscopic lesions on endoscopy and (2) histologic detection of CMV infection in GI tissue. Histologic detection of CMV infection in GI tissue was defined as (1) identification of CMV inclusion bodies on hematoxylin and eosin staining, (2) positive immunohistochemical staining for CMV, and/or (3) positive CMV polymerase chain reaction (PCR) results. Tissue specimens were obtained with forceps biopsy during esophagogastroduodenoscopy, enteroscopy, sigmoidoscopy, and/or colonoscopy. On the basis of these criteria, a total of 86 immunocompetent patients with CMV gastroenterocolitis were included in the final analysis of this study.

### Data collection

We reviewed the medical records and endoscopy images of the 86 immunocompetent patients diagnosed with CMV gastroenterocolitis. The clinical data at the presentation of CMV gastroenterocolitis were investigated, which included age, sex, body mass index, underlying comorbid diseases, and symptoms. American Society of Anesthesiologists (ASA) class was assessed for the evaluation of the general physical condition of patients. In addition, laboratory findings, if available, including fecal *Clostridium difficile* toxin, serum CMV DNA, and serum CMV immunoglobins (Ig)M and IgG antibodies, were investigated. Endoscopic images were independently evaluated by two endoscopists (JYY and JSB). After a meticulous review of endoscopy images and discussion between the two endoscopists, we classified the endoscopic features of CMV gastroenterocolitis into a discrete ulcerative type with or without exudate and a diffuse erythematous type with or without exudate. In this study, exudate was defined as a pseudomembrane-like, thick, inflammatory exuded semi-fluid matter covering the lesion. Finally, the types of treatment and clinical course of the patients were also analyzed. Death was defined as in-hospital mortality during admission. This study was carried out in accordance with STROBE guidelines and Declaration of Helsinky. The study protocol was approved by the institutional review board of Asan medical Center, Seoul, Korea (approval no. 2018-0643). Informed consent was waived from the institutional review board of Asan medical Center due to the retrospective design.

### Statistical analysis

Pearson’s chi-square test was used to compare categorical variables expressed as numbers with percentages. Student’s t-test was used to compare continuous variables presented as mean ± standard deviation. Univariable and multivariable logistic regression analyses were performed for risk factor investigation by estimating the odds ratios (ORs). Variables with *P* values < 0.2 from univariable analysis were included in multivariable analysis. A *P* value of < 0.05 was considered statistically significant. All data were analyzed using R statistical package version 3.6.0 (R Foundation for Statistical Computing, Vienna, Austria).

## Supplementary Information

Supplementary Information.

## Data Availability

All authors agreed to make materials, data and associated protocols promptly available to readers without undue qualifications in material transfer agreements.

## References

[1] Lachance P, Chen J, Featherstone R, Sligl WI (2017). Association between cytomegalovirus reactivation and clinical outcomes in immunocompetent critically ill patients: A systematic review and meta-analysis. Open Forum Infect. Dis..

[2] You DM, Johnson MD (2012). Cytomegalovirus infection and the gastrointestinal tract. Curr. Gastroenterol. Rep..

[3] Galiatsatos P, Shrier I, Lamoureux E, Szilagyi A (2005). Meta-analysis of outcome of cytomegalovirus colitis in immunocompetent hosts. Dig. Dis. Sci..

[4] Iwamuro M (2017). Endoscopic manifestations and clinical characteristics of cytomegalovirus infection in the upper gastrointestinal tract. Acta Med Okayama.

[5] Grilli E, Galati V, Bordi L, Taglietti F, Petrosillo N (2012). Cytomegalovirus pneumonia in immunocompetent host: Case report and literature review. J. Clin. Virol..

[6] Joye A, Gonzales JA (2018). Ocular manifestations of cytomegalovirus in immunocompetent hosts. Curr. Opin. Ophthalmol..

[7] Waqas QA, Abdullah HMA, Khan UI, Oliver T (2019). Human cytomegalovirus pneumonia in an immunocompetent patient: A very uncommon but treatable condition. BMJ Case Rep..

[8] Le PH (2017). Clinical characteristics of cytomegalovirus colitis: A 15-year experience from a tertiary reference center. Ther. Clin. Risk Manag..

[9] Rafailidis PI, Mourtzoukou EG, Varbobitis IC, Falagas ME (2008). Severe cytomegalovirus infection in apparently immunocompetent patients: A systematic review. Virol. J..

[10] Al-Omari A, Aljamaan F, Alhazzani W, Salih S, Arabi Y (2016). Cytomegalovirus infection in immunocompetent critically ill adults: Literature review. Ann. Intensive Care.

[11] Siciliano RF, Castelli JB, Randi BA, Vieira RD, Strabelli TM (2014). Cytomegalovirus colitis in immunocompetent critically ill patients. Int. J. Infect. Dis..

[12] Frydrych LM, Bian G, O'Lone DE, Ward PA, Delano MJ (2018). Obesity and type 2 diabetes mellitus drive immune dysfunction, infection development, and sepsis mortality. J. Leukoc. Biol..

[13] Syed-Ahmed M, Narayanan M (2019). Immune dysfunction and risk of infection in chronic kidney disease. Adv. Chronic Kidney Dis..

[14] Schmucker DL, Heyworth MF, Owen RL, Daniels CK (1996). Impact of aging on gastrointestinal mucosal immunity. Dig. Dis. Sci..

[15] Ko JH (2015). Clinical presentation and risk factors for cytomegalovirus colitis in immunocompetent adult patients. Clin. Infect. Dis..

[16] Chaemsupaphan T (2020). Patient characteristics, clinical manifestations, prognosis, and factors associated with gastrointestinal cytomegalovirus infection in immunocompetent patients. BMC Gastroenterol..

[17] Seo TH (2012). Cytomegalovirus colitis in immunocompetent patients: A clinical and endoscopic study. Hepatogastroenterology.

[18] Bernard S (2015). Symptomatic cytomegalovirus gastrointestinal infection with positive quantitative real-time PCR findings in apparently immunocompetent patients: A case series. Clin. Microbiol. Infect..

