# Supplementary material for: Endoscopic features and clinical outcomes of cytomegalovirus gastroenterocolitis in immunocompetent patients
Source: Sci Rep. 2021 Mar 18;11:6284. doi: 10.1038/s41598-021-85845-8 (PMC7973552; doi:10.1038/s41598-021-85845-8)
Supplement: Supplementary file 1 — Supplementary Information. [file 41598_2021_85845_MOESM1_ESM.doc]

**Supporting information**

**Endoscopic features and clinical outcomes of cytomegalovirus gastroenterocolitis in immunocompetent patients**

Jiyoung Yoon^*^, Junghwan Lee^*^, Dae Sung Kim, Jin Wook Lee, Seung Wook Hong, Ha Won Hwang, Sung Wook Hwang, Sang Hyoung Park, Dong-Hoon Yang, Byong Duk Ye, Seung-Jae Myung, Hwoon-Yong Jung, Suk-Kyun Yang & Jeong-Sik Byeon^‡^

**Table of Contents**

1. **Supplementary Figure ……………………………………………….………….……2**
2. **Supplementary Table ……………………………………………….………….……3**


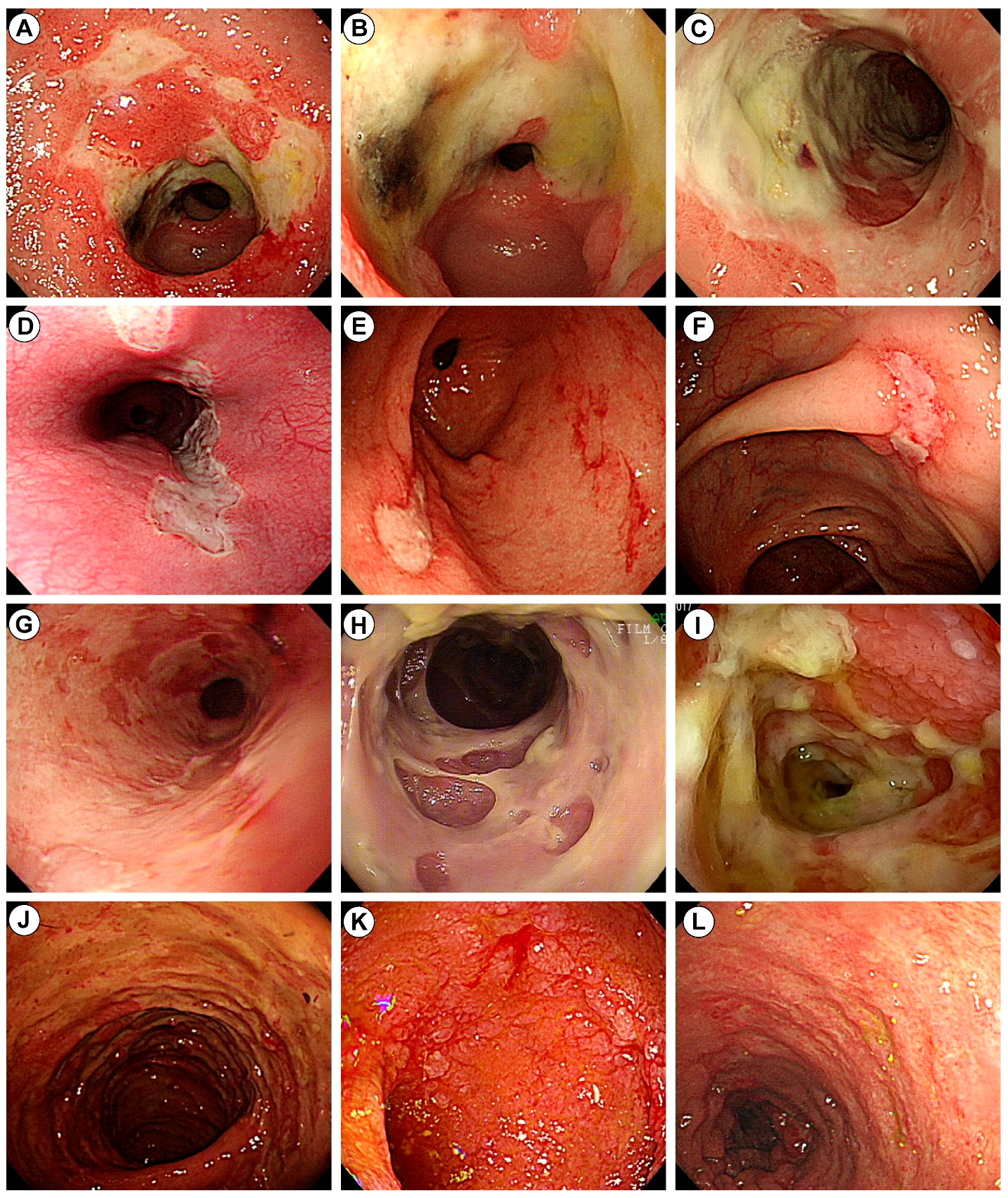


**Supplementary Figure 1.** Endoscopic images of typical cytomegalovirus gastroenterocolitis in immunocompetent patients. (A–C) Discrete ulcerative type with exudate. A and B, stomach; C, colon. (D–F) Discrete ulcerative type without exudate. D, esophagus; E, stomach; F, colon. (G–J) Diffuse erythematous type with exudate. G, esophagus; H and I, small bowel; J, colon. (K–L) Diffuse erythematous type without exudate. K, small bowel; L, colon.

**Supplementary Table 1.** Risk factors for surgery in immunocompetent patients with cytomegalovirus gastroenterocolitis

|  | Univariable analysis | | | Multivariable analysis | |
| --- | --- | --- | --- | --- | --- |
|  | OR (95% CI) | | *P* value | OR (95% CI) | *P* value |
| Age (< 60 vs. ≥ 60 years) | 1.05 (0.94–1.27) | | 0.234 |  |  |
| Male sex | 1.65 (0.22–12.28) | | 0.627 |  |  |
| BMI^a^ | 0.88 (0.65–1.20) | | 0.434 |  |  |
| ASA class | 1.81 (0.68–4.83) | | 0.238 |  |  |
| Location |  | |  |  |  |
| UGI-dominant group | ꝏ | | 1.000 |  |  |
| SB-dominant group | ꝏ | | 1.000 |  |  |
| Colon-dominant group | ꝏ | | 1.000 |  |  |
| Laboratory findings | |  |  |  |  |
| Hemoglobin | 0.82 (0.46–1.48) | | 0.515 |  |  |
| CRP | 1.14 (1.03–1.29) | | 0.044* | 1.22 (1.05–1.41) | 0.009* |
| Albumin | 1.04 (0.26–4.11) | | 0.959 |  |  |
| Endoscopic types |  | |  |  |  |
| Discrete ulcer  with exudate or without exudate | 1.00 | |  |  |  |
| Discrete ulcer  with or without exudate | 0.58 (0.06–5.81) | | 0.641 |  |  |
| Other risk factors |  | |  |  |  |
| Ongoing infection | 4.45 (0.44–44.68) | | 0.204 |  |  |
| ICU stay | 1.56 (0.21–11.66) | | 0.663 |  |  |
| Antiviral therapy | 0.67 (0.09–5.02) | | 0.699 |  |  |

OR, odds ratio; CI, confidence interval; BMI, body mass index; ASA, American Society of Anesthesiologists; UGI, upper gastrointestinal tract; SB, small bowel; CRP, C-reactive protein; ICU, intensive care unit

^a^BMI data were not available for 2 of the 86 patients
